# Identification of Dual-Target Inhibitors for Epidermal Growth Factor Receptor and AKT: Virtual Screening Based on Structure and Molecular Dynamics Study

**DOI:** 10.3390/molecules28227607

**Published:** 2023-11-15

**Authors:** Hanyu Yang, Zhiwei Zhang, Qian Liu, Jie Yu, Chongjin Liu, Wencai Lu

**Affiliations:** 1 College of Physics, Qingdao University, Qingdao 266071, China; 2020025451@qdu.edu.cn (H.Y.); 2020025442@qdu.edu.cn (Z.Z.); 2019020188@qdu.edu.cn (J.Y.); 2020020330@qdu.edu.cn (C.L.); 2 School of Medicine and Pharmacy, Ocean University of China, Qingdao 266003, China; lq6360@stu.ouc.edu.cn

**Keywords:** small molecule inhibitors, EGFR, AKT, TCMIO, HMDB

## Abstract

Epidermal growth factor EGFR is an important target for non-small cell lung (NSCL) cancer, and inhibitors of the AKT protein have been used in many cancer treatments, including those for NSCL cancer. Therefore, searching small molecular inhibitors which can target both EGFR and AKT may help cancer treatment. In this study, we applied a ligand-based pharmacophore model, molecular docking, and MD simulation methods to search for potential inhibitors of EGFR and then studied dual-target inhibitors of EGFR and AKT by screening the immune-oncology Chinese medicine (TCMIO) database and the human endogenous database (HMDB). It was found that TCMIO89212, TCMIO90156, and TCMIO98874 had large binding free energies with EGFR and AKT, and HMDB0012243 also has the ability to bind to EGFR and AKT. These results may provide valuable information for further experimental study.

## 1. Introduction

The epidermal growth factor receptor (EGFR) is a transmembrane glycoprotein with an extracellular EGF binding domain and an intracellular tyrosine kinase domain. It plays a crucial role in regulating signaling pathways and controlling cellular proliferation. EGFR has been found to be overexpressed in many cancer cells [1]. This overexpression leads to autophosphorylation of its tyrosine residues, triggering a cascade of events that further affect cell proliferation, differentiation, and apoptosis [2]. EGFR overexpression has been observed in several types of cancer, including non-small cell lung cancer, pancreatic cancer, glial cell carcinoma, and other tissues. The inhibition of EGFR overexpression is considered a crucial strategy in current anti-cancer research [1]. Current studies on EGFR inhibitors can be divided into two main categories: monoclonal antibodies (mAb) and small molecule tyrosine kinase inhibitors (TKIs) [3]. EGFR inhibitors have emerged as a crucial class of antitumor drugs. The discovery of highly effective EGFR inhibitors is of paramount importance in the treatment of tumors, including lung cancer, cervical cancer, and breast cancer [4,5]. At present, a large number of small molecule TKIs targeting EGFR have been developed [6,7,8,9,10].

Gefitinib (IC50 = 23–79 nM) and Erlotinib (IC50 = 80 nM) have been clinically used as first-generation representative EGFR-TKIs compared to standard chemotherapy [11]. These drugs act as reversible competitive inhibitors of ATP to prevent EGFR autophosphorylation [12]. In the treatment of breast cancer, Afatinib (IC50 = 0.5 nM), a second-generation EGFR-TKI, has gained significant popularity [13,14,15]. In addition, a third-generation TKI called Osimertinib (IC50 = 12 nM) specifically targets EGFR-T790M resistance mutations that occur due to the use of first-generation TKIs [16,17].

In the field of natural product inhibitors of EGFR, research has shown that sesquiterpene lactones, chalcone, and phenolic compounds can be synergistically combined with small molecule inhibitors to enhance drug sensitivity and improve therapeutic outcomes [18]. Abou-Zied HA et al. developed a novel xanthine derivative that incorporates the chalcone component for investigating potential EGFR inhibitors and successfully obtained compound **11**, which exhibited an IC50 value of 0.3 µM against the target enzyme [19]. Abdelgawad et al. developed innovative phenolic compounds as potential inhibitors of EGFR and COX-2. Their research resulted in the identification of compounds C4 and G4, which demonstrated impressive inhibitory activity with IC50 values of 0.9 and 0.5 µM, respectively [20]. Furthermore, an interesting study conducted by N Nerdy et al. revealed that sesquiterpene lactones derived from veronica amygdaline defile exhibited potential anticarcinogenic effects through the inhibition of EGFR expression [21].

However, despite the potent activity demonstrated by these EGFR inhibitors, challenges such as drug resistance and weak efficacy in cells and in vivo settings persist [22]. Therefore, there is an urgent necessity for the development of novel EGFR inhibitors. This urgency arises from the fact that the activation of EGFR profoundly impacts a multitude of signaling mechanisms within cells. As the downstream protein of EGFR, AKT assumes a crucial role in regulating cell proliferation and survival. Moreover, AKT acts as a constitutively active kinase that promotes the survival of NSCLC cells [23]. Notably, the heightened activity of AKT has been associated with the persistence of drug resistance against first-generation EGFR TKIs [24,25].

The computational study of drugs plays an important role in the field of drug discovery and has gained widespread usage in identifying potential new drug candidates. The strategy of screening large virtual molecular libraries uses a series of methodologies, including molecular docking, the construction of pharmacodynamic groups, and the application of quantitative structure–activity relationships (QSARs). These approaches have been effectively employed in the quest for identifying inhibitors targeting EGFR or AKT [26,27,28]. Currently, there is an increasing emphasis on the research of protein inhibitors that target dual or even multiple targets. Alessia Bono et al. conducted a study on a novel dual-target inhibitor for SARS-CoV-2 MPRO, resulting in the identification of six potential compounds [29]. Similarly, Edgar et al. utilized the MD method to investigate dual inhibitors for G9a and DNMT1, successfully indentifying compounds exhibiting dual activity [30]. However, there have been very few computational studies for dual-target inhibitors of EGFR and AKT. Tarozzi A et al. undertook the design and investigation of several quinazoline derivatives to study EGFR and AKT dual inhibitors [31]. In this study, our objective was to search for dual inhibitors targeting EGFR and AKT in the traditional Chinese medicine on immune-oncology (TCMIO) and human endogenous library (HMDB) databases [32,33] using a pharmacophore model, molecular docking, and molecular dynamics (MD) simulation methods. The identified inhibitor candidates can potentially contribute valuable insights for further experimental investigations on novel inhibitors targeting EGFR and AKT.

## 2. Results and Discussion

### 2.1. Results of Pharmacophore and Molecular Docking Screening

**Figure 1:**
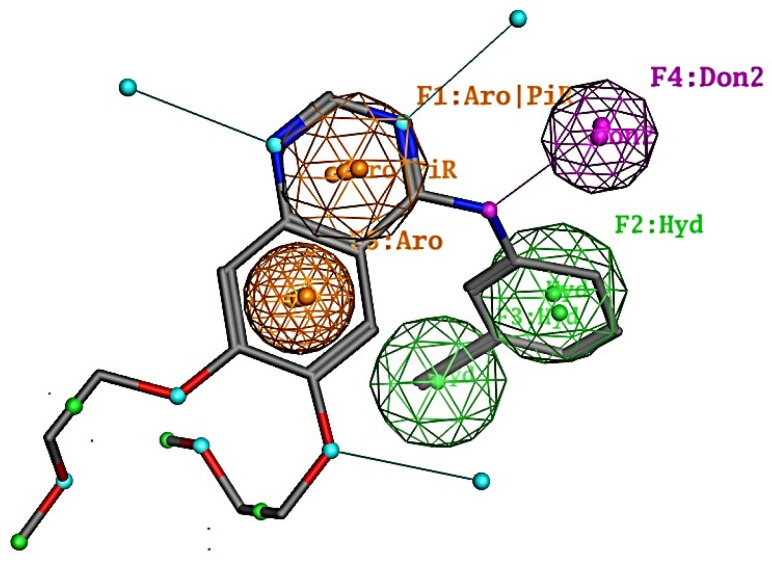
Pharmacophore with the F1-F5 features constructed from 20 EGFR positive compounds and its overlap with Erlotinib. Green: Hydrophobic centroid. Purple: H-bond donor. Orange: Aromatic center.

A set of 20 inhibitor compounds, each possessing distinct active structures, was employed to construct pharmacophore models. Finally, a pharmacophore model with two aromatic rings, two hydrophobicity characteristics, and one H-bond donor characteristic (Figure 1) was selected based on the overlap and accuracy values.

Using the developed pharmacophore model, a comprehensive screening process was carried out on a total of 240,956 compounds from the TCMIO and HMDB datasets. 3252 compounds successfully passed the screening criteria, and, subsequently, the top 20% of compounds (456 in TCMIO and 194 in HMDB) were selected baed on ranking the RMSD (root mean square deviation) scores.

**Figure 2:**
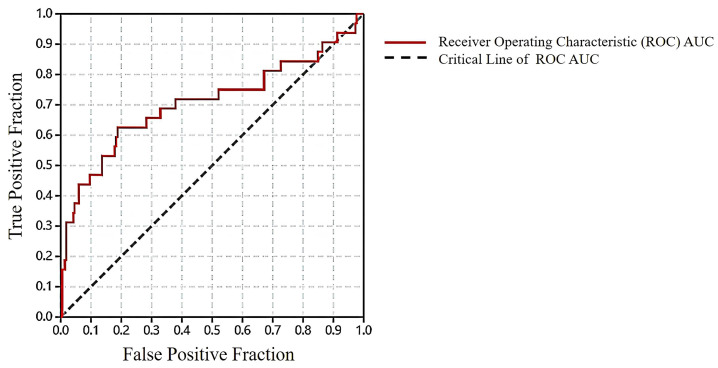
ROC verification of molecular docking method.

**Figure 3:**
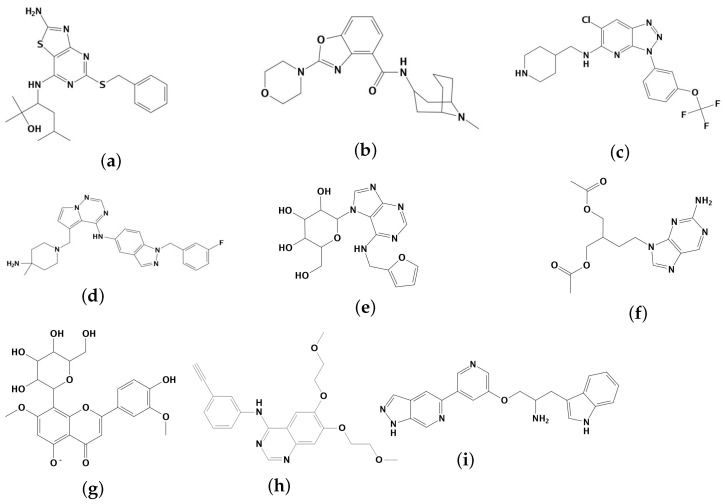
Two-dimensional structures of (**a**) TCMIO5312, (**b**) TCMIO89212, (**c**) TCMIO90156, (**d**) TCMIO98874, (**e**) HMDB0012243, (**f**) HMDB0014570, (**g**) HMDB0037450, (**h**) Erlotinib, and (**i**) A-443654.

**Figure 4:**
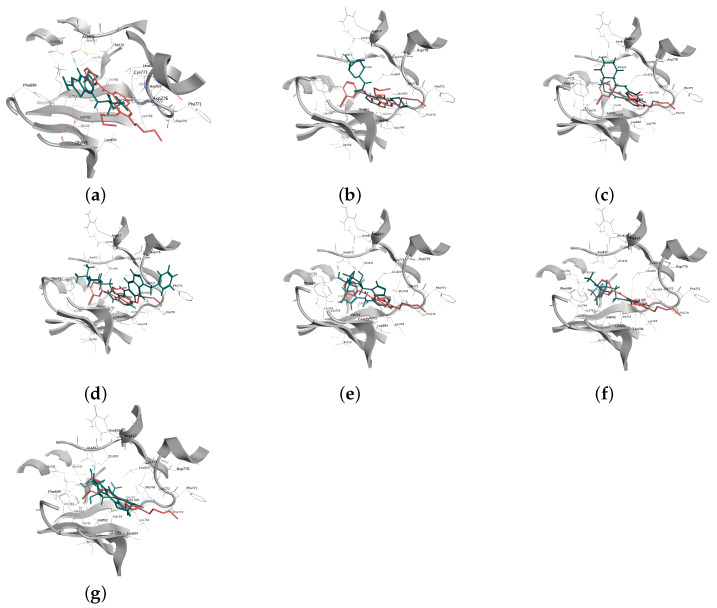
Three-dimensional docking structures with EGFR of the candidate inhibitors (green) (**a**) TCMIO5312, (**b**) TCMIO89212, (**c**) TCMIO90156, (**d**) TCMIO98874, (**e**) HMDB0012243, (**f**) HMDB0014570, (**g**) HMDB0037450, and Erlotinib (red).

In order to ascertain the accuracy and reliability of molecular docking, the receiver operating characteristic (ROC) curve for the validation index was calculated based on the true positive rate and false positive rate. The resulting area under the curve (AUC) value was determined to be 0.707 (Figure 2). Furthermore, the docking result of protein inhibitors showed that the RMSD value of Erlotinib was 1.35 Å, and its docking score of the best conformation was −7.44 kcal/mol. Similarly, the AKT ligand, A-443654, demonstrated an RMSD of 1.43 Å, with a corresponding docking score of −8.42 kcal/mol. The ROC validation results and the docking results of the inhibitors from EGFR and AKT demonstrated the reliability of the molecular docking method. Based on the analysis of docking results, it was observed that the selected compounds were capable of effectively binding to the ligand-binding sites of EGFR and AKT. Finally, a total of seven compounds with the best docking scores were chosen for further analyses (Figure 3). Met769 is the key residue of EGFR inhibitors (such as Erlotinib), contributing to protein structure stabilization or functional regulation. It interacts with the hinge region of 4-aniline quinazoline scaffold through specific binding interactions [34]. Table 1 shows that the compounds TCMIO89212, TCMIO90156, TCMIO98874, HMDB0014570, and HMDB0037450 formed H-bond interactions with the key residue Met769 of EGFR. Some of these compounds also formed hydrogen bonds with the residues Asp831, Gln767, Asn818, Thr830, and Lys721 (Table 1). A previous study by Yang et al. reported that the key residue Asp831 is involved in H-bond interactions [35]. It was suggested that strong interactions between compounds and Asp831 played an important role in inhibiting EGFR activity [36]. The docking results shown in Table 1 indicated that TCMIO5312, TCMIO98874, HMDB0012243, and HMDB0037450 formed H-bond interactions with Asp831. As depicted in Figure 4, the compounds were successfully docked in the binding pocket of EGFR.

**Figure 5:**
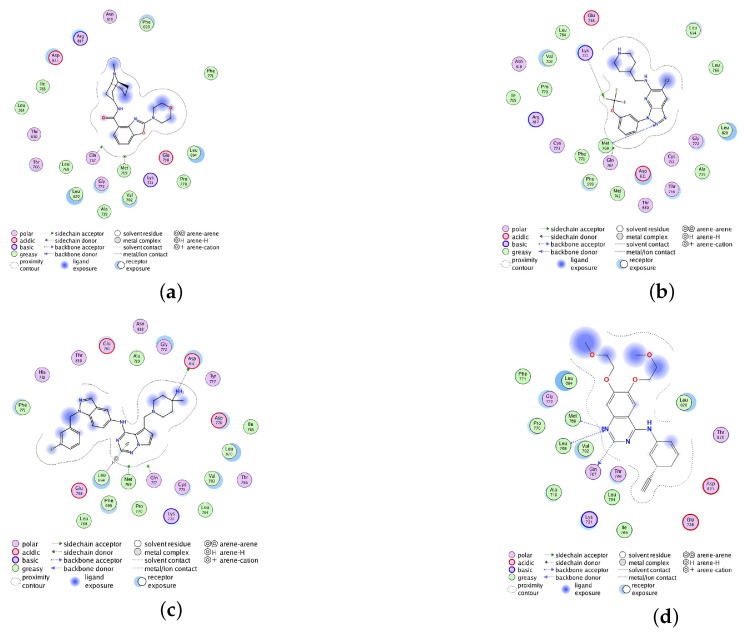
Schematic 2D representations of the interactions between EGFR and (**a**) TCMIO89212, (**b**) TCMIO90156, (**c**) TCMIO98874, and (**d**) Erlotinib, respectively.

The docking scores of TCMIO98874, TCMIO90156, and TCMIO89212 with EGFR were −7.96, −7.51, and −7.06 kcal/mol, respectively. Figure 4 shows the 3D docking structures with EGFR for all compounds compared to Erlotinib. The 2D interactions of TCMIO98874, TCMIO90156, TCMIO89212, and Erlotinib docking to the EGFR ligand-binding site are shown in Figure 5, in which Erlotinib formed H-bond interactions with Met769, Leu768, and Gln767. On the other hand, TCMIO89212, TCMIO98874, and TCMIO90156 also formed H-bond interactions with Met769. Interestingly, in addition to its interactions with Met769, TCMIO98874 also formed H-bond interactions with Asp831.

**Figure 6:**
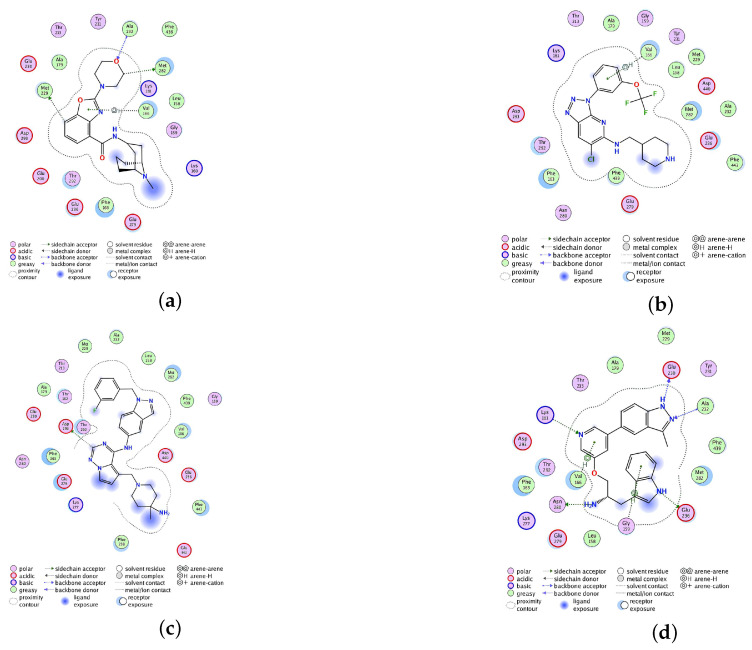
Schematic 2D representations of the interactions between AKT and (**a**) TCMIO89212, (**b**) TCMIO90156, (**c**) TCMIO98874, and (**d**) A-443654, respectively.

In addition, according to the docking results of the seven compounds with AKT (Table 2), TCMIO90156, TCMIO89212, and TCMIO98874 exhibited docking scores of −8.28, −8.24, and −8.00 kcal/mol, respectively. The docking score of inhibitor A-443654 was calculated to be −8.42 kcal/mol. As indicated in Table 2, A-443654 demonstrated H-bond interactions with Glu230, Asn280, Asp290, Ala232, and Lys181 at the AKT binding site. Additionally, it exhibited an electrostatic interaction with Asp293. On the other hand, TCMIO89212 formed H-bond interactions with Met229, Met282, and Ala332 (Figure 6). TCMIO90156 primarily interacted with Glu236 at the AKT binding site, while TCMIO98874 predominantly interacted with Asp440. TCMIO90156 exhibited a predomonant interaction with Glu236 in AKT, while TCMIO98874 primarily interacted with Asp440 in AKT.

### 2.2. MD Simulation Results

Using Amber22, we performed MD simulations on the complexes of seven compounds (TCMIO5312, TCMIO89212, TCMIO 90156, TCMIO98874, HMDB0012243, HMDB-0014570, and HMDB0037450) and the inhibitors of Erlotinib and A-443654 with EGFR and AKT. The analysis focused on the root mean square deviation (RMSD), root mean square fluctuation (RMSF), rotation radius (Rg), and hydrogen bonds formed during the simulation [37].

During the MD simulations, the ligands were initially placed at the binding site of the target protein based on molecular docking. Taking TCMIO90156 as an example, the 100 ns MD simulations were analyzed to observe the conformation changes of the ligand and target protein. Figure S1 showed that the composite structure of the protein with TCMIO90156 was gradually compacted during 100 ns. The protein structure underwent a gradual tightening process within the initial <20 ns timeframe. After a 20 ns MD run, the amplitude of protein structural changes appeared to be relatively small. Furthermore, the conformational changes occurring at the ligand–protein binding site did not exhibit noticeable variations during the 90 to 100 ns MD simulations, and TCMIO90156 became stabilized within the binding pocket.

#### 2.2.1. RMSD, RMSF, and Rg Analyses

**Figure 7:**
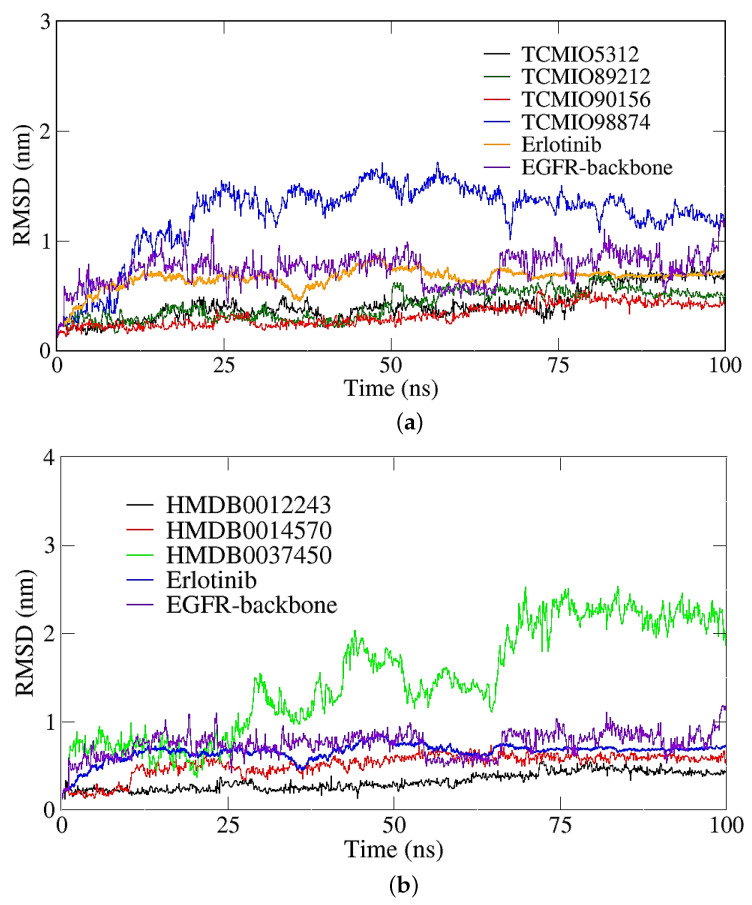
Backbone RMSD of the complexes of EGFR and inhibitor candidates screened from (**a**) TCMIO and (**b**) HMDB databases.

RMSD values obtained during MD simulations serve as indicators of conformational changes in receptor–ligand complexes [38]. As shown in Figure 7, it is apparent that the RMSD values of all the EGFR–compound complexes increased rapidly at the beginning of the MD simulations. Subsequently, the RMSD value for each complex in Figure 7 exhibited fluctuations within the range of 0.50 to 2.50 nm, suggesting the stability of these complexes. Notably, TCMIO90156, which possessed the most favorable binding free energy, achieved stabilization after 75 ns of simulation.

**Figure 8:**
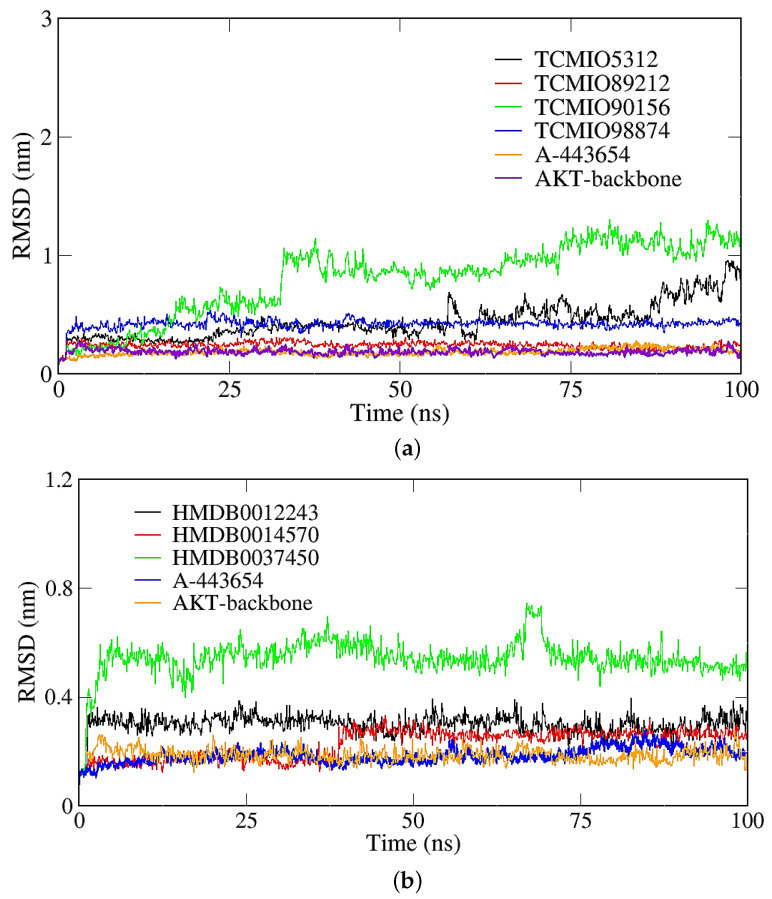
Backbone RMSD of the complexes of AKT and inhibitor candidates screened from (**a**) TCMIO and (**b**) HMDB databases.

Figure 8 shows the RMSD results of the complex structures of seven compounds with AKT. In comparison to the control A-443654, the RMSD vaues for TCMIO90156, TCMIO5312, and HMDB0037450 demonstrated significant fluctuations, while the remaining compoounds exhibited minimal fluctuations and were stabilized below 0.50 nm. During the 100 ns MD, the RMSD of TCMIO90156 experienced a rapid increase to 1 nm at 30 ns. However, the RMSD of TCMIO5312 gradually stabilized after 75 ns. As for HMDB0037450, its RMSD increased between 68 and 70 ns, reaching 0.7 nm, then decreased to 0.6 nm and remained stable. These observations suggest that all seven complexes of AKT remained stable throughout the MD simulations.

**Figure 9:**
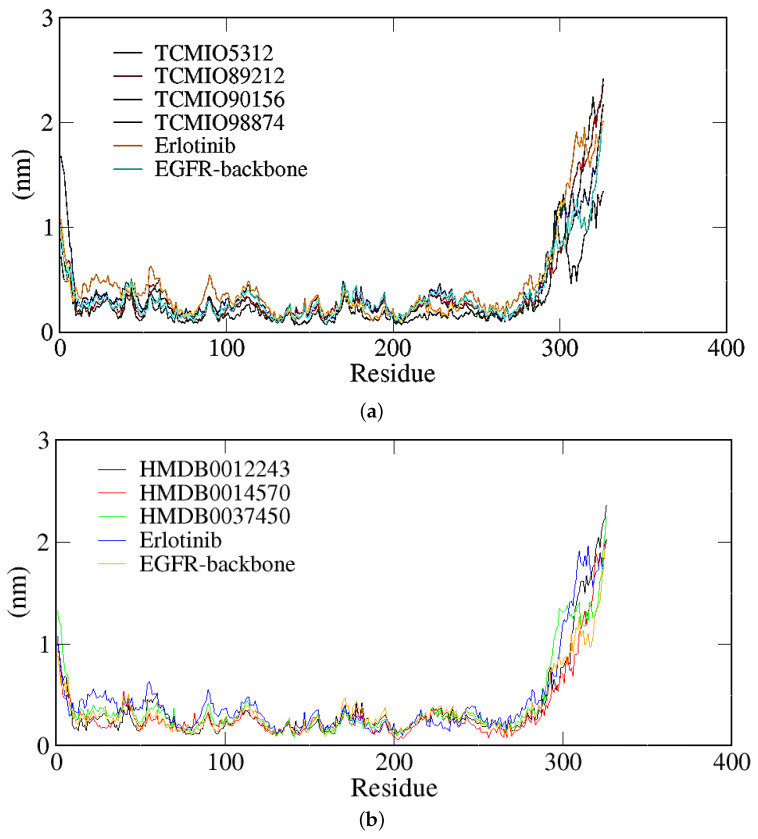
RMSF of the complex structures of EGFR with the TCMIO (**a**) and HMDB (**b**) compounds.

The RMSF values serve as indicators of the flexibility of complex structures. A higher RMSF value indicates greater flexibility [39]. In general, the terminus regions of protein structures exhibited higher flexibility as depicted in Figure 9. The majority of residues demonstrated similar RMSF values within the EGFR complex structures, suggesting that the binding of the compound to EGFR did not noticeably affect the stability of the protein structure.

It can be seen from Figure S2 that the RMSF values of the compounds bound to AKT were comparable to those of the EGFR complexes. Most residues exhibited RMSF values below 0.5 nm. Among the seven compounds, the average fluctuations of all compounds, except for TCMIO89212 and TCMIO90156, were lower than those of the control A-443654.

**Figure 10:**
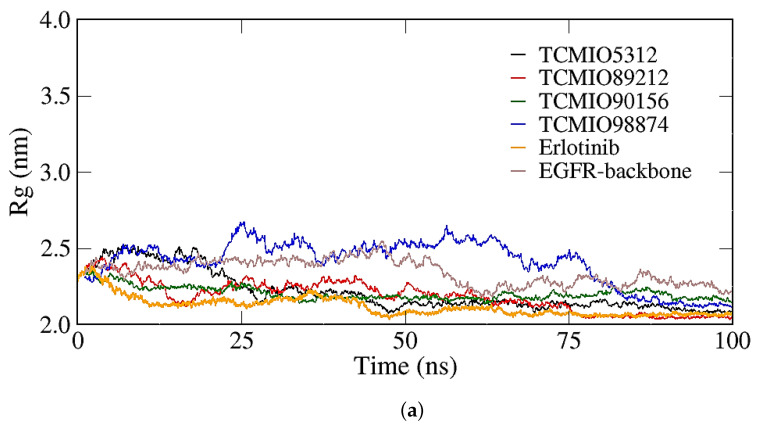

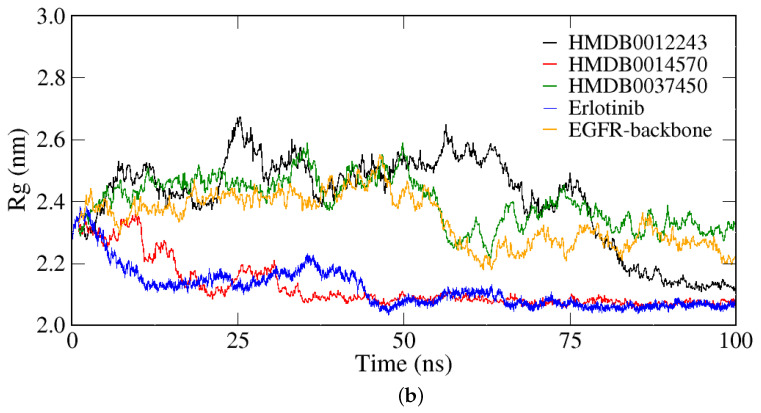
Radii of gyration (Rg) of the complex structures of EGFR with the TCMIO (**a**) and HMDB (**b**) compounds.

The radii of Gyration (Rg) values were employed to assess the folding rate and the sizes of protein–compound complexes. Rg serves as a critical measure of protein compactness, with higher Rg values indicating lower protein compactness [39]. During the MD simulations, the Rg values were assessed. As depicted in Figure 10, it is evident that the Rg values fluctuated due to the inherent dynamism of the complexes. However, all the Rg values remained within the range of 2.0–2.80 nm, suggesting the absence of significant conformational changes. The complexes formed with TCMIO90156, TCMIO89212, TCMIO98874, TCMIO5312, HMDB0012243, and HMDB0037450 were observed to exhibit tighter structures compared with the Erlotinib complex. This observation suggests that these six compounds had a lesser impact on the tightness of the protein [40].

The AKT complexes analyzed in Figure S3 displayed Rg values ranging from 1.9 to 2.5 nm, suggesting the absence of noteworthy replaced conformational changes. Notably, all the compounds, except for TCMIO89212 and TCMIO90156, exhibited fluctuations around the 2 nm mark.

#### 2.2.2. Analyses of H-Bonds

**Figure 11:**
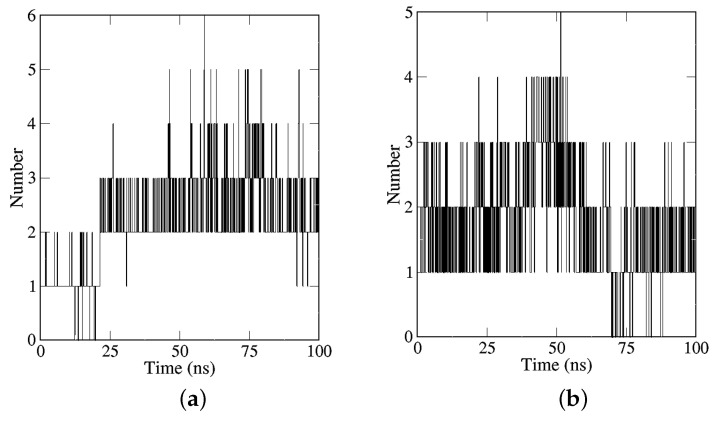

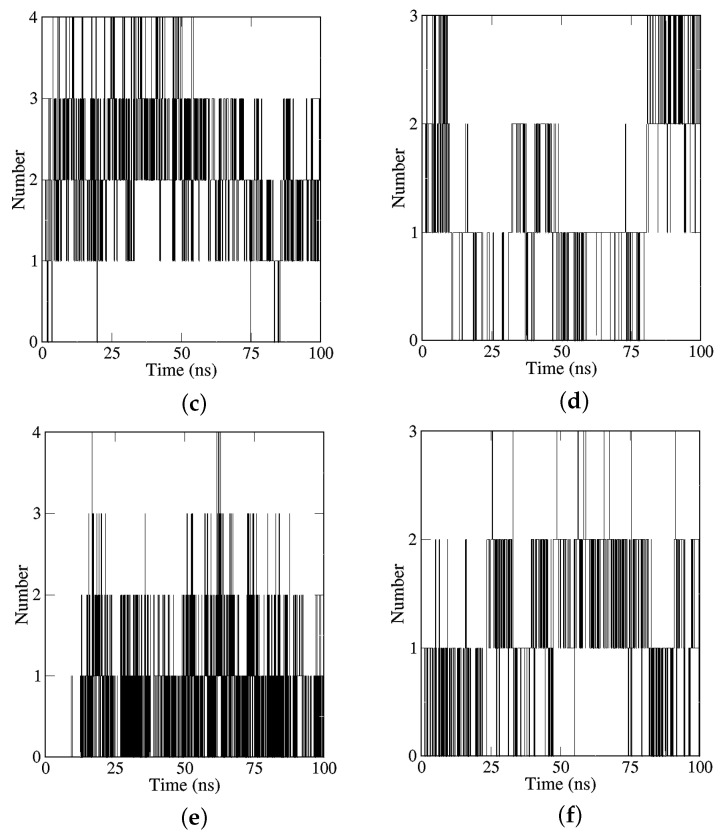
Numbers of intermolecular hydrogen bonds of (**a**) TCMIO98874, (**b**) TCMIO5312, (**c**) TCMIO90156, (**d**) TCMIO89212, (**e**) Erlotinib, and (**f**) EGFR-backbone binding to EGFR from 100 ns MD simulations.

The H-bond module was utilized to determine the number of H-bonds formed between the ligand and receptor throughout the course of the MD simulations [41]. In general, it is expected that a ligand can establish at least two H-bonds with the receptor protein. Upon analyzing the H-bond count for three compounds in relation to EGFR, it is observed that the reference compound (Erlotinib) formed a maximum of four H-bonds during the 100 ns simulations. However, TCMIO98874, TCMIO5312, TCMIO90156, and TCMIO89212 were able to form a maximum of six, five, four, and three H-bonds with EGFR, respectively (Figure 11). As H-bond interactions are crucial in intermolecular binding, it was anticipated that TCMIO98874, TCMIO5312, and TCMIO90156 would exhibit efficient binding with EGFR [42].

Figure S4 showed that TCMIO98874, TCMIO90156, and TCMIO89212 have the potential to form a maximum of eight, six, and three H-bonds, respectively, with AKT. In comparison, the control compound A-443654 formed five hydrogen bonds.

#### 2.2.3. Binding Free Energy Analyses

The MM-PBSA binding free energies were calculated using gmx_MMPBA [43]. The energy components, including van der Waals, electrostatic, polar solvation, and solvent accessible surface area (SASA) and total binding free energy, for the seven compounds are provided in Table 3.

From Table 3, it is evident that the binding free energies of TCMIO90156 were lower than those of the control compound Erlotinib. The binding free energies of TCMIO90156, TCMIO98874, TCMIO5312, and TCMIO89212 were all lower compared with the binding free energy of EGFR-backbone. This suggests that these compounds may have positive effects on EGFR. Notably, the lowest binding energy observed for TCMIO90156 (−35.74 kcal/mol) was nearly three times higher than that of HMDB0037450 (−13.05 kcal/mol). This significant disparity in binding free energies might align with the substantial distinction in electrostatic energies between these two compounds. The results showed that TCMIO90156, TCMIO98874, TCMIO5312, and TCMIO89212, especially TCMIO90156, could potentially exhibit a potent inhibitory effect on EGFR.

According to the binding free energies of the complexes with AKT (Table 4), the binding free energy of the inhibitor A-443654 (−38.37 kcal/mol) was higher compared with those of TCMIO98874 (−56.32 kcal/mol), TCMIO89212 (−33.18 kcal/mol), and TCMIO90156 (−29.83 kcal/mol). Based on the estimated binding free energies, TCMIO98874 appeared to have a higher binding affinity for AKT compared with A-443654. Additionally, the binding free energy of HMDB0012243 was observed to be −21.68 kcal/mol, which is comparatively lower than those of the other HMDB compounds listed in Table 4. Consequently, TCMIO98874, TCMIO89212, and TCMIO90156 have been identified as potential dual inhibitors of EGFR and AKT, based on the aforementioned findings.

#### 2.2.4. Analysis of the Interaction between Ligands and Proteins

**Figure 12:**
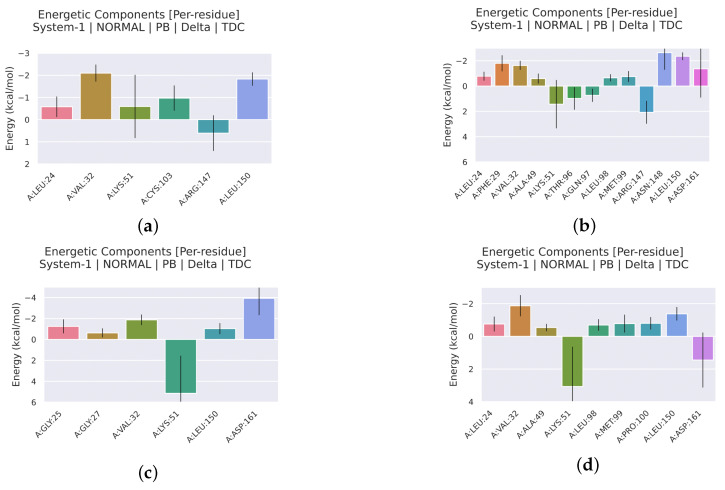
MMPBSA energy residue decomposition analysis of (**a**) TCMIO89212, (**b**) TCMIO90156, (**c**) TCMIO98874, and (**d**) Erlotinib with EGFR.

**Figure 13:**
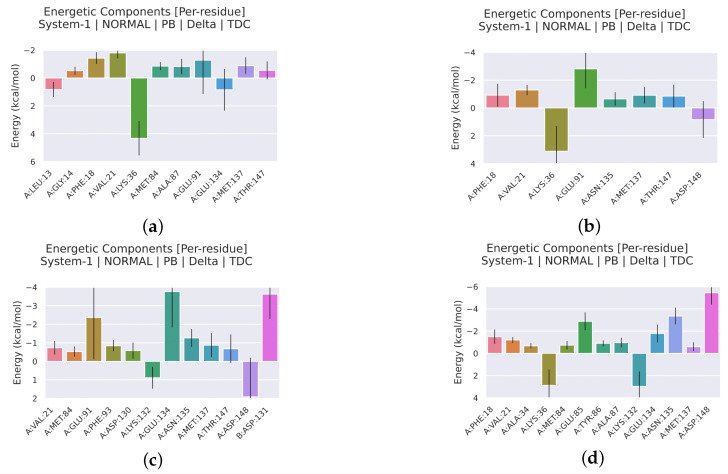
MMPBSA energy residue decomposition analysis of (**a**) TCMIO89212, (**b**) TCMIO90156, (**c**) TCMIO98874, and (**d**) A-443654 with AKT.

Interaction energy decomposition analyses were performed for the protein–ligand complexes involving TCMIO89212, TCMIO90156, and TCMIO98874 with both EGFR and AKT. The results of these analyses can be observed in (Figure 12 and Figure 13). Figure 12 demonstrates that Erlotinib exhibited robust interactions with the residues Val32 and Leu150 of EGFR. On the other hand, in the case of TCMIO89212, TCMIO90156, and TCMIO98874, the binding energies primarily relied on the contributions from different residue pairs: Val32 and Leu150; Asn148 and Leu150; and Asp161 and Val32, respectively. These findings shed light on the key residues involved in the binding interactions between these ligands and EGFR.

**Figure 14:**
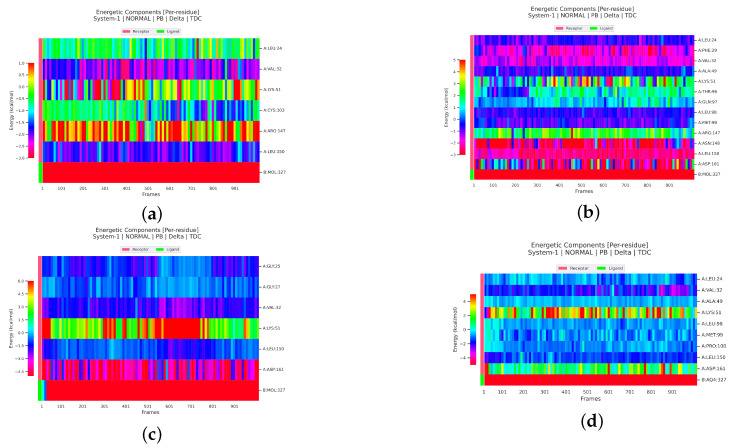
Analysis of MMPBSA energy residue decomposition heatmaps of (**a**) TCMIO89212, (**b**) TCMIO90156, (**c**) TCMIO98874, and (**d**) Erlotinib with EGFR. Different colors represent different energy values, and the more negative energy values in each row, the stronger the interaction between the corresponding residues of that rowand small molecules.

**Figure 15:**
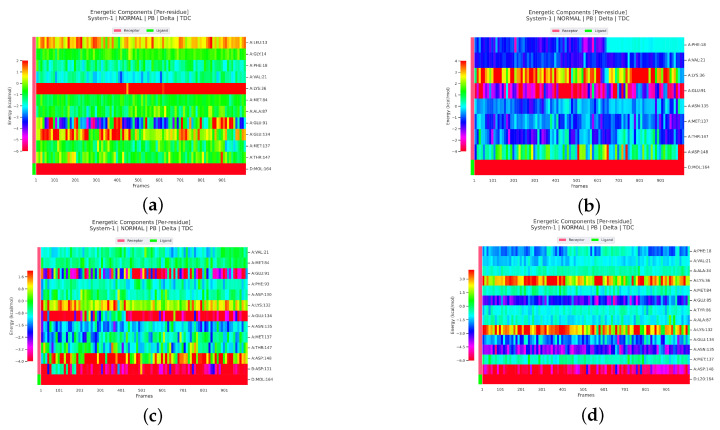
Analysis of MMPBSA energy residue decomposition heatmaps of (**a**) TCMIO89212, (**b**) TCMIO90156, (**c**) TCMIO98874, and (**d**) A-443654 with AKT. Different colors represent different energy values, and the more negative energy values in each row, the stronger the interaction between the corresponding residues of that rowand small molecules.

Figure 14 and Figure 15 illustrate the energy contributions of the crucial EGFR and AKT residues throughout the MD simulation preocesses involving TCMIO89212, TCMIO90156, and TCMIO98874. The MD simulations showed that key residues in the Erlotinib–EGFR complex, Val32 and Leu150 with TCMIO89212, Asn148 and Leu150 with TCMIO90156, and Asp161 and Val32 with TCMIO98874 maintained crutial interactions throughout the MD simulation processes. This suggests that these residues are important for the molecular recognition and binding of these ligands with EGFR. During the MD simulations of AKT, it was observed that the key residue interactions of Asp148, Asn135, and Glu85 with the positive drug A-443654, Glu91 with TCMIO89212, Glu91 with TCMIO90156, and Glu134 and Glu91 with TCMIO98874 were consistently maintained.

Figure 15 reveals that the positive drug A-443654 exhibited stronger interactions with Asp148, Asn135, and Glu85 of AKT. As for TCMIO89212, TCMIO90156, and TCMIO98874, the residues Val21, Phe18, and Glu91; Glu91; and Glu134 and Glu91, respectively, significantly contributed to the binding energies.

#### 2.2.5. ADMET Profiling

**Figure 16:**
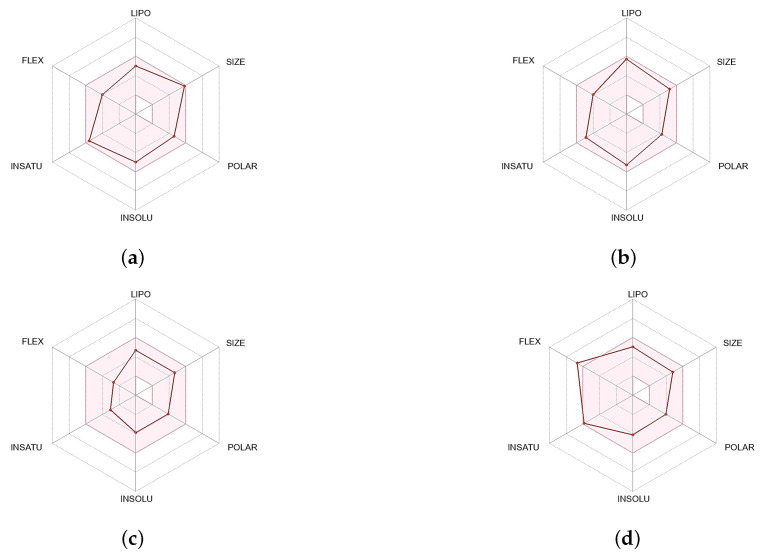
ADMET analyses of (**a**) TCMIO90156, (**b**) TCMIO89212, (**c**) TCMIO98874, and (**d**) Erlotinib. The color area is the suitable physicochemical space for oral bioavailability. The line represent the various parameter values predicted by the platform for compounds.

ADMET analyses were conducted for TCMIO90156, TCMIO89212, and TCMIO98874. The pink region depicted in Figure 16 represents the optimal range for each characteristic, with lipophiles (XLOGP3) spanning from −0.7 to +5.0. The size varied from 150 to 500 g/mol, the polar TPSA spanned from 20 to 130 Å2, the solubility (LogS) ranged from −6 to 0, and the unsaturation fraction ranged from 0.25 to 1. The ADMET prediction results (Figure 16) indicated that all three compounds satisfied the restriction criteria of ADMET, suggesting a lower potential for toxicity risk.

## 3. Screening Methods

### 3.1. Pharmacophore and Molecular Docking

MOE 2022 was used to construct a pharmacophore model with an active coverage of 0.9, query spacing of 0.6, query cluster of 1.25, feature number of 5, and default values for the remaining parameters. For the generation of pharmacophore models, a training set consisting of 20 known EGFR inhibitors with diverse structures was obtained from the BindingDB database [44], and the IC50 values of these inhibitors ranged between 0.13 nmol/L and 1000 nmol/L. Finally, a pharmacophore model containing three characteristics, aromatics (AROs), an H-bond donor (Don2), and hydrophobicity (Hyd), was constructed (Figure 1). Virtual screenings using the pharmacophore model were performed to search for inhibitor candidates in the TCMIO database (126,973 compounds) and HMDB database (113,983 compounds). The pharmacophore model successfully filtered out a total of 3252 compounds.

Subsequently, molecular docking screenings were conducted using MOE 2022 with the application of an Amber10: ETH force field. During the molecular docking process, the receptor was set as rigid, and for each compound docking, a total of 5 optimal compound conformations were generated; the GBVI/WSA dG method [45] was used to score compound postures. The structures of EGFR (PDB 1M17) and AKT (PDB 2JDR) were employed in this study, and were prepared by retaining the protein conformations, simultaneously using Homology Builder in MOE 2022 to complete missing protein residues and adding hydrogens. Following the pharmacophore screening, the 3252 compounds obtained were sorted based on the RMSD values, and 650 compounds of top 20% were chosen for molecular docking at the ligand-binding site of EGFR. In order to verify the docking method’s effectiveness, both the test set and decoy set were subjected to the pharmacophore screening and docking with EGFR. A receiver operating characteristic ROC curve analysis was employed to assess the docking screening’s capability to distinguish active compounds. Models with an area under the curve AUC value ⩾ 0.7 or greater were considered to be possibly reliable [46].

The test set comprised 32 active compounds specifically targeting EGFR. This set included 20 carefully chosen inhibitors sourced from the BindingDB database. Its purpose was to assess the predictive ability of molecular docking. Additionally, a decoy set consisting of 218 inactive compounds was generated using Dude (http://dude.docking.org/ (accessed on 16 December 2022)) in order to evaluate the efficacy of the molecular docking [47]. The decoy set was carefully assembled to consist of the compounds that share similar physical and chemical properties with the active set but no pharmacological activity [48,49]. The primary objectives was to develop a pharmacophore model which will be able to detect compounds within an active/test set, while effectively minimizing the inclusion of decoy compounds. The validation process of molecular docking screening involved the utilization of both the test and decoy sets. This validation was conducted by calculating the cumulative area under the curve (AUC) of the receiver operating characteristic (ROC) using various statistical indicators. The compounds exhibiting superior docking scores, which were lower than those of the inhibitors, were carefully chosen. Furthermore, the compounds selected based on the pharmacophore model and molecular docking of EGFR were subjected to further docking analysis within the AKT ligand-binding site. In this analysis, an AKT inhibitor was used as a reference.

### 3.2. Molecular Dynamics (MD) Simulations

All MD simulations were conducted employing Amber22 [50], an ff19SB force field was utilized for protein parameterization [51], and a GAFF2 force field was applied to handle compound interactions [52]. All MD simulations were run with a TIP3P water model [53,54]. The solvation simulation of receptor–ligand complex was conducted under the periodic boundary condition using a dodecahedron periodic box. The TIP3P water model was employed for solvation.To maintain charge balance in the system, Na${}^{+}$ and Cl${}^{-}$ ions were added. Subsequently, the equilibrium phase, production simulation, and MMPBSA calculations were performed using the AmberMDrun command [55]. The ten-step simulation preparation scheme is a widely-accepted equilibrium method for solvated biomolecules. It is applicable to various types of systems and allows for simple tests based on system density to ensure simulation stability. This simulation approach has been extensively validated and has shown to be effective and versatile, having been successfully applied to diverse protein and nucleic acid systems [56]. Constant temperature was controlled with a velocity rescaling thermostat [57], and constant pressure was controlled with a Berendsen barostat [58].The system was initially equilibrated at 300 K through 100 ps NVT and NPT simulations, respectively, and the reference pressure for the NPT ensemble was set to 1 bar.

The 100 ns MD simulations with a time step of 2 fs were performed for the complexes of the selected compounds with EGFR and AKT added. The gmx_MMPBSA tool was utilized to estimate the binding energy of each complex by analyzing the MD trajectory [59]. This tool calculates the binding energy based on a total of 100 frames extracted from the 100 ns production simulation trajectory.

### 3.3. ADMET Evaluation

The structures of the compounds were converted into the simplified molecular input line input system (SMILES) format. Subsequently, the documents were submitted to http://www.swissadme.ch/ (accessed on 7 March 2023) SwissADME [60] to evaluate the metabolism, excretion, distribution, toxicity, and absorption characteristics of small molecules.

## 4. Conclusions

We employed a ligand-based pharmacophore filtration and molecular docking screening approach to identify potential natural compound inhibitors within the TCMIO database, as well as endogenous compounds in the HMDB. These compounds were targed to simultaneously inhibit both EGFR and AKT. A total of seven compounds were discovered, demonstrating a strong alignment with the pharmacophore model and exhibiting favorable docking scores. Among them, TCMIO90156 demonstrated the lowest binding free energy when compared with the control compound Erlotinib for EGFR. Additional, the binding free energies of TCMIO98874, TCMIO5312, and TCMIO89212 were in close proximity to the binding free energy of Erlotinib. The results obtained from the MD simulation indicated that the binding free energy of TCMIO98874 with AKT was lower compared with the control compound A-443654. On the other hand, the binding free energies of TCMIO90156 and TCMIO89212 were slightly higher than that of A-443654. The analysis of their interactions with EGFR showed that these compounds exhibited similar interaction patterns with the reference drugs of EGFR. Furthermore, MD simulations indicated that TCMIO90156, TCMIO89212, and TCMIO98874 displayed relatively low binding free energies and formed crucial bindings with the essential residues of both EGFR and AKT. These findings suggest that these compounds have the potential to function as dual-target inhibitors, simultaneously targeting both EGFR and AKT. The findings of this study offer valuable insights for further experimental inverstigations. The goal moving forward is develop a web-based platform that provides a user-friendly dataset of EGFR inhibitors, as described in article [61]. This will serve as a valuable resource for researchers in the field.

## Figures and Tables

**Table 1:** GBVI/WSA dG docking scores of the EGFR–ligand complexes.

Compound ID	Docking Score (kcal/mol)	Interaction Type	Key Amino Acid	Ligand Efficiency (kcal/mol)
TCMIO5312	−7.72	H-donor	Glu831, Met742,	−0.276
			Asp831	
		H-acceptor	Asp831	
TCMIO89212	−7.06	H-acceptor	Met769, Gln767	−0.252
TCMIO90156	−7.51	H-acceptor	Met769, Lys721,	−0.256
			Gln767	
		H-acceptor	Met769	
TCMIO98874	−7.96	H-donor	Asp831	−0.221
		H-acceptor	Met769, Gln767	
HMDB0012243	−7.29	H-donor	Asp831	−0.270
HMDB0014570	−7.33	H-acceptor	Met769	−0.318
HMDB0037450	−7.85	H-donor	Asp776, Gln767	−0.231
		H-acceptor	Met769, Thr830,	
			Asp831, Lys721	
		Ionic	Lys721	
Erlotinib	−7.37	H-acceptor	Met769	−0.254

**Table 2:** GBVI/WSA dG docking scores of the AKT–ligand complexes.

Compound ID	Docking Score (kcal/mol)	Interaction Type	Key Amino Acid	Ligand Efficiency (kcal/mol)
TCMIO5312	−8.08	H-donor	Asn820, Glu236	−0.289
		Ionic	Glu279	
TCMIO89212	−8.24	H-donor	Met229, Met282	−0.294
		H-acceptor	Ala232	
TCMIO90156	−8.28	H-donor	Glu236	−0.285
		H-donor	Asp831	
TCMIO98874	−8.00	H-donor	Asp440	−0.222
		Ionic	Asp440	
HMDB0012243	−8.17	H-donor	Met229	
		H-acceptor	Ala232, Asp293	
			Lys181	
HMDB0014570	−8.15	H-donor	Glu230	−0.354
		H-acceptor	Asp293, Ala232	
HMDB0037450	−9.32	H-donor	Glu230, Gln236	−0.274
		H-acceptor	Lys181, Glu236,	
			Asp440	
		Ionic	Lys181	
A-443654	−8.42	H-donor	Glu230, Asn280,	−0.280
			Asp290, Glu236	
		H-acceptor	Ala232, Lys181	
		Ionic	Asp293	

**Table 3:** MM-PBSA binding free energies and energy components (kcal/mol) of the complexes of EGFR and screened TCMIO and HMDB compounds.

Ligands	ΔEvanderWaal	ΔEelectrostatic	ΔEpolarSolvation	SASA	ΔGbinding
TCMIO90156	−47.01	−131.48	147.32	−4.57	−35.74
TCMIO98874	−29.94	−243.41	247.05	−3.36	−29.66
TCMIO5312	−36.35	−146.94	160.09	−4.45	−27.65
TCMIO89212	−42.79	−149.06	169.92	−4.27	−26.20
HMDB0012243	−36.89	−49.90	70.77	−4.12	−20.14
HMDB0014570	−40.65	−16.51	45.41	−3.92	−15.67
HMDB0037450	−15.32	−49.39	54.12	−2.45	−13.05
Erlotinib	−47.65	−146.57	167.75	−4.32	−30.79
EGFR-backbone	−33.10	−14.21	29.35	−3.60	−21.57

**Table 4:** MM-PBSA binding free energies and energy components (kcal/mol) of the complexes of AKT and screened TCMIO and HMDB compounds.

Ligands	ΔEvanderWaal	ΔEelectrostatic	ΔEpolarSolvation	SASA	ΔGbinding
TCMIO98874	−52.28	−647.65	648.81	−5.20	−56.32
TCMIO89212	−52.31	−262.09	285.63	−4.41	−33.18
TCMIO90156	−31.11	−310.42	312.20	−3.50	−29.83
TCMIO5312	−29.42	−261.69	275.08	−3.88	−19.91
HMDB0012243	−35.02	−61.14	78.35	−3.87	−21.68
HMDB0014570	−42.16	−29.37	54.73	−3.88	−20.68
HMDB0037450	−36.89	−74.53	111.81	−4.39	−4.00
A-443654	−42.52	−270.18	278.73	−4.4	−38.37
AKT-backbone	−42.98	−274.20	276.02	−4.43	−45.59

## Data Availability

Data are contained within the article and Supplementary Materials.

## Supplementary Materials

The following supporting information can be downloaded at: https://www.mdpi.com/article/10.3390/molecules28227607/s1, Figure S1: ADMET analyze of TCMIO5312, HMDB0012243, HMDB0014570, HMDB0037450; Figure S2: RMSF of AKT complexes; Figure S3: Rg of AKT complexes; Figure S4: H-bond analyses of EGFR complexes (a) TCMIO5312, (b) HMDB0012243, (c) HMDB0037450, (d) HMDB0014570; Figure S5: H-bond analyses of AKT complexes H-bond analyses of AKT complexes (a) TCMIO5312, (b) TCMIO89212, (c) TCMIO90156, (d) TCMIO98874, (e) HMDB0012243, (f) HMDB0037450, (g) HMDB0014570, (h) A-443654; Table S1. The full list of the active ligands used.

## Abbreviations

The following abbreviations are used in this manuscript:

$\Delta E_{SASA}$	Solvent accessible surface area
