# Supplementary material for: Identification of Dual-Target Inhibitors for Epidermal Growth Factor Receptor and AKT: Virtual Screening Based on Structure and Molecular Dynamics Study
Source: Molecules. 2023 Nov 15;28(22):7607. doi: 10.3390/molecules28227607 (PMC10673407; doi:10.3390/molecules28227607)
Supplement: Supplementary file 1 [file molecules-28-07607-s001.zip › molecules-2641447-supplementary.pdf]

Supplementary Material

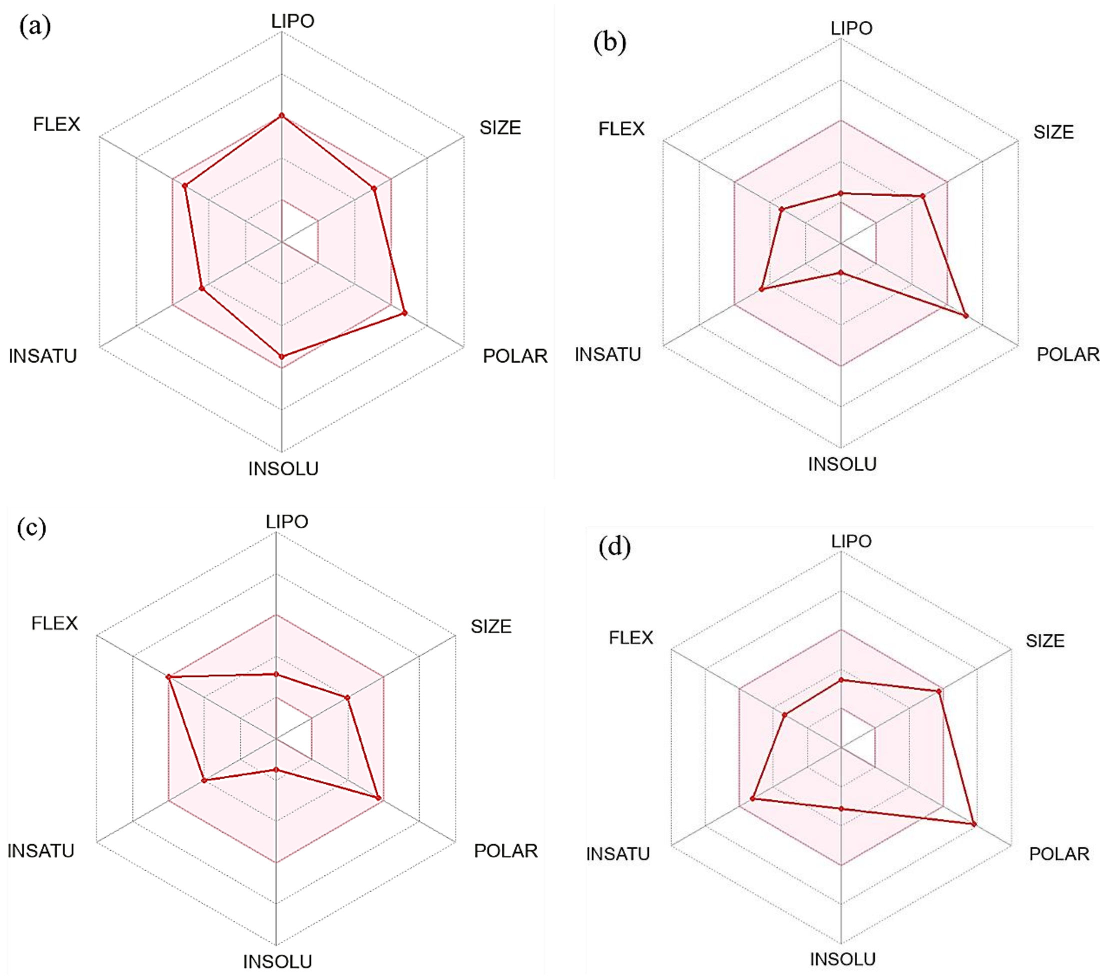

Figure S1. ADMET analyze of TCMIO5312, HMDB0012243, HMDB0014570, HMDB0037450.

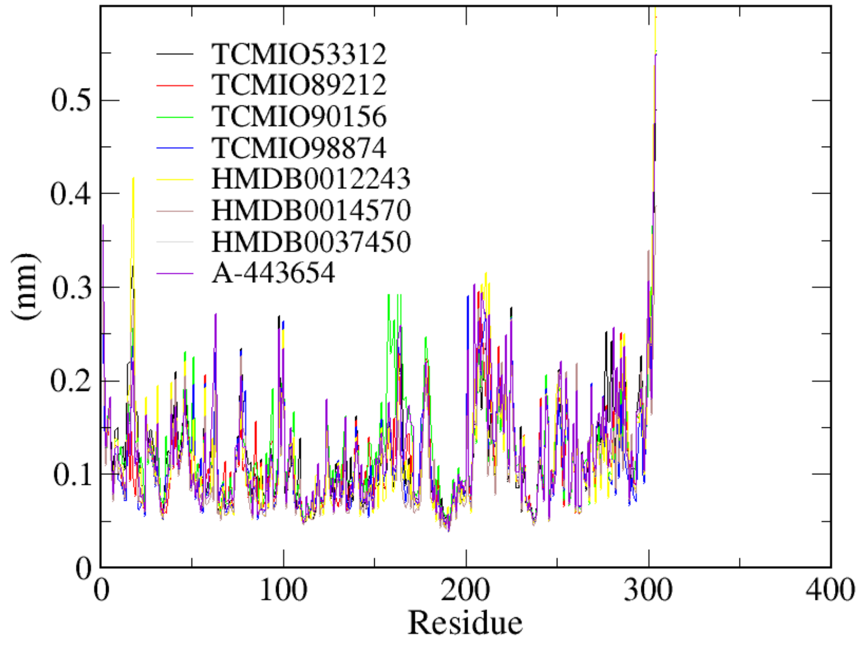

Figure S2. RMSF of AKT complexes:

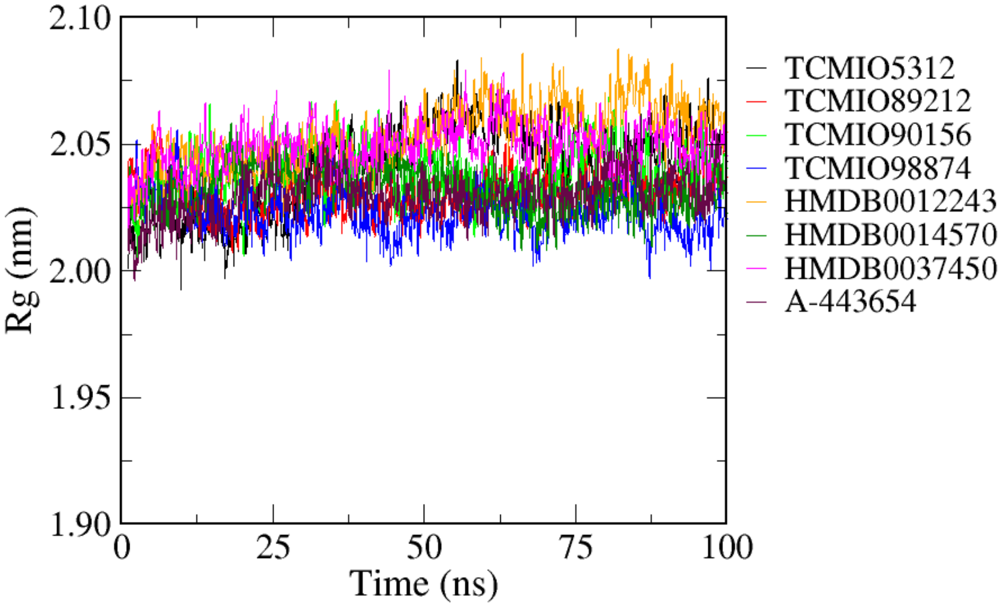

Figure S3. Rg of AKT complexes

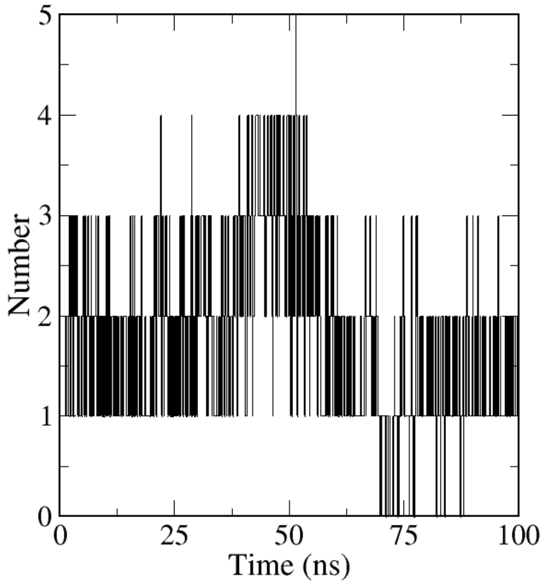

(a)

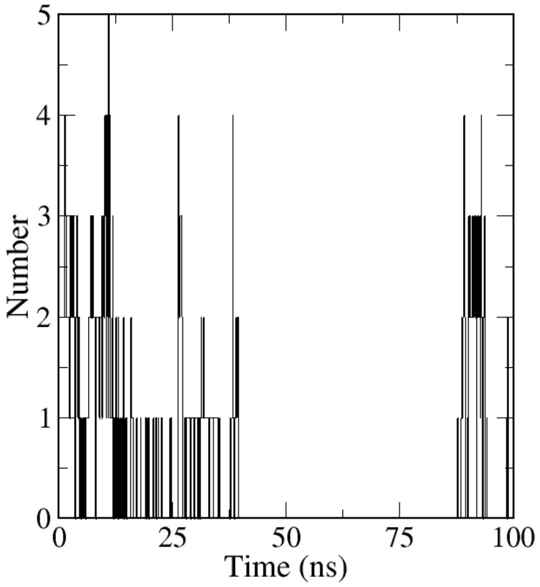

(b)

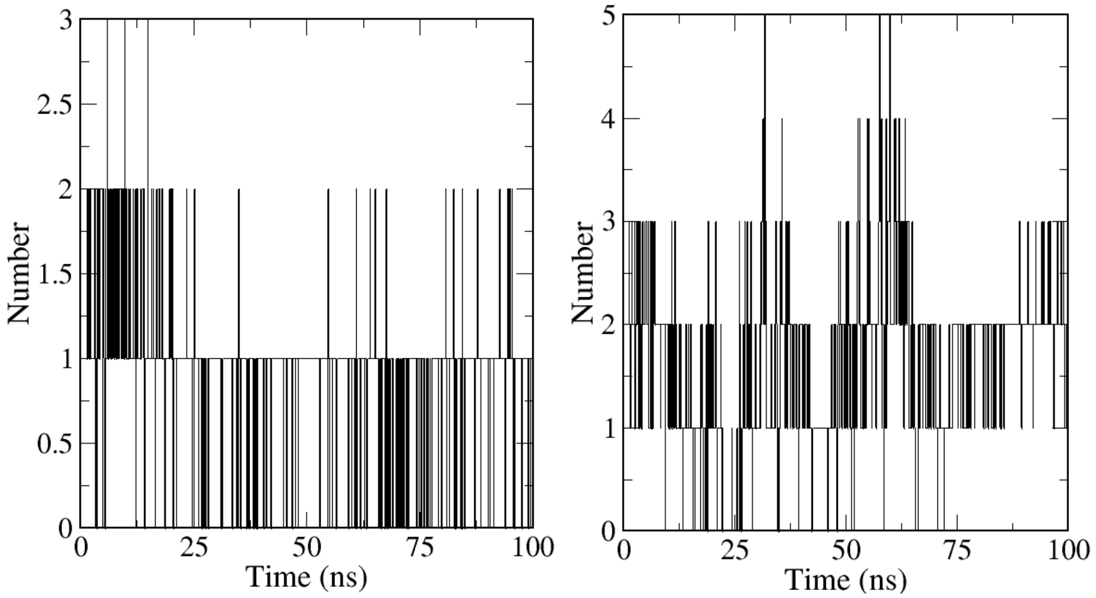

(c) (d)

Figure S4. H-bond analyses of EGFR complexes (a) TCMIO5312, (b) HMDB0012243, (c) HMDB0037450, (d) HMDB0014570.

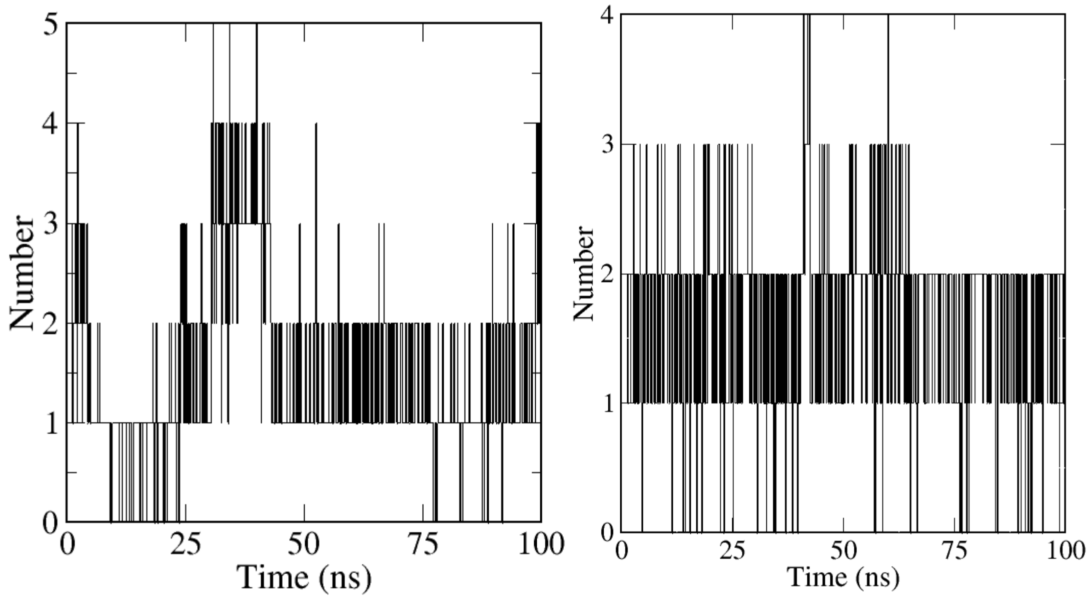

(a) (b)

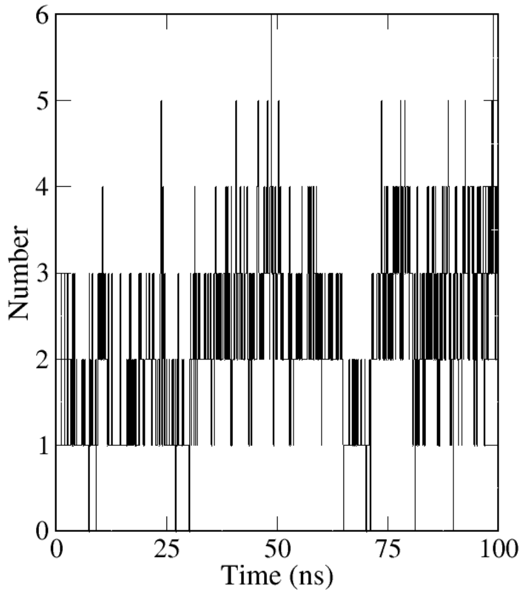
6
5
4
3
2
1
0
Number
0
25
50
75
100
Time (ns)

(c)

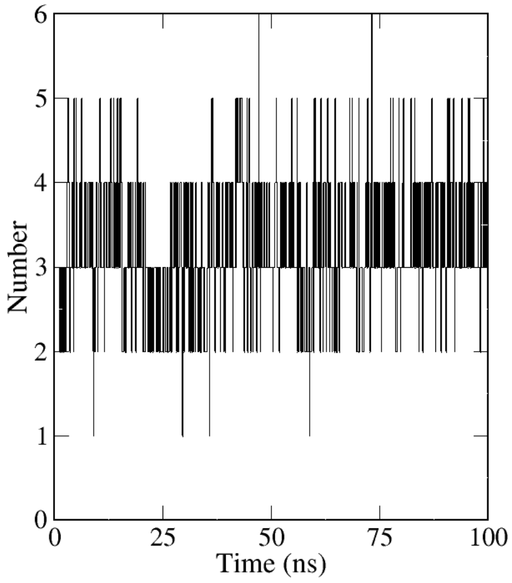
6
5
4
3
2
1
0
Number
0
25
50
75
100
Time (ns)

(d)

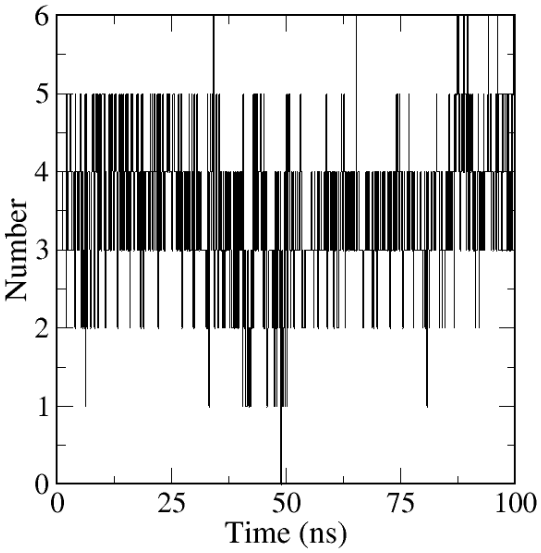
6
5
4
3
2
1
0
Number
0
25
50
75
100
Time (ns)

(e)

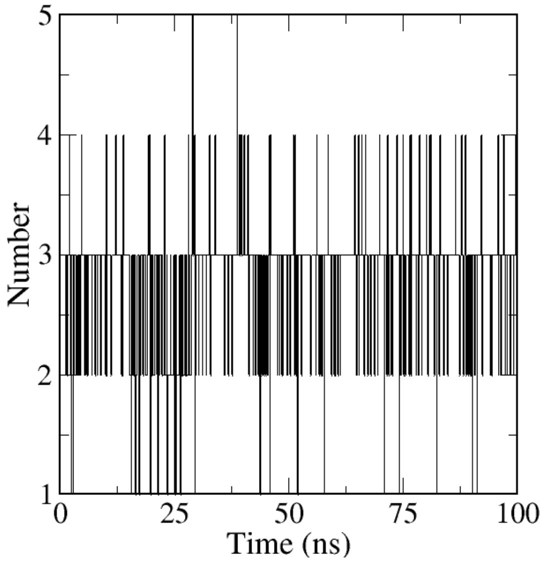
5
4
3
2
1
Number
0
25
50
75
100
Time (ns)

(f)

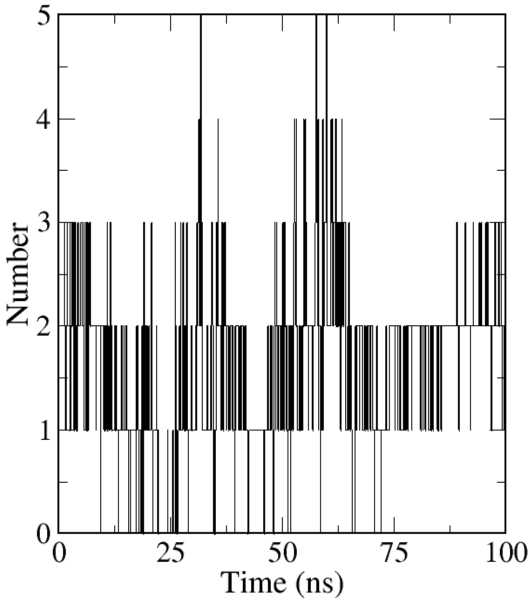

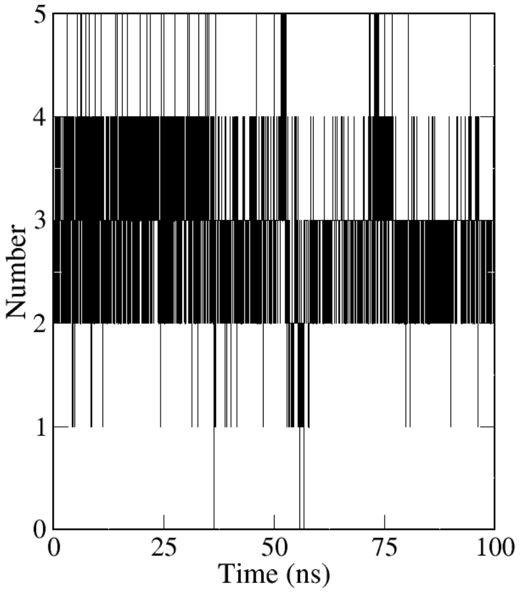

(g) (h)

Figure S5. H-bond analyses of AKT complexes H-bond analyses of AKT complexes (a) TCMIO5312, (b) TCMIO89212, (c) TCMIO90156, (d) TCMIO98874, (e) HMDB0012243, (f) HMDB0037450, (g) HMDB0014570, (h) A-443654.

Table S1. The full list of the active ligands used

num	mol	Structure	IC50(um)
1	5328245	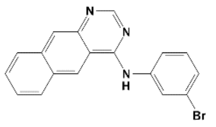	0.003
2	2857	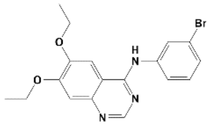	0.006
3	122658923	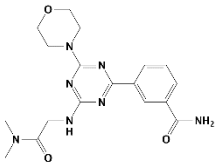	0.007
4	4705	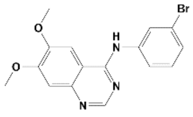	0.029
5	5328253	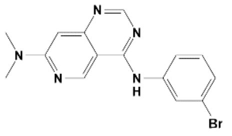	0.091

6	5328045	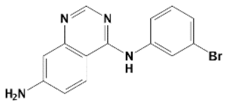	0.1
7	2426	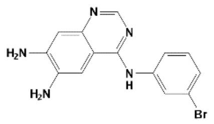	0.12
8	10320940	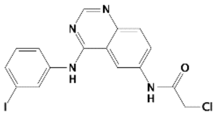	0.12
9	5328258	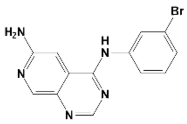	0.13
10	123631	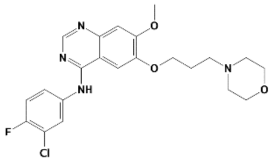	0.15
11	10528818	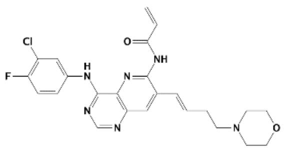	0.16
12	135402838	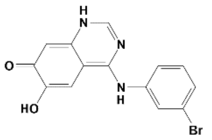	0.17
13	5328221	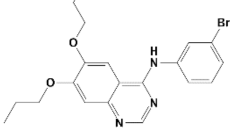	0.174
14	5328352	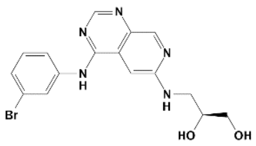	0.18
15	5328373	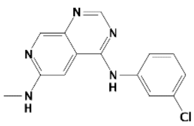	0.19
16	5280961	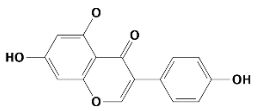	0.2

17	5328202	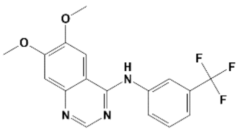	0.245
18	5328342	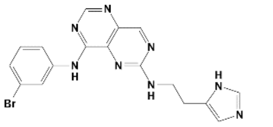	0.25
19	5328409	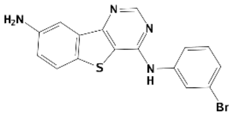	0.27
20	5328233	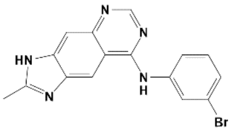	0.29
